# Anticandidal Activity of *Asparagus racemosus*

**DOI:** 10.4103/0250-474X.56017

**Published:** 2009

**Authors:** B. Uma, K. Prabhakar, S. Rajendran

**Affiliations:** Department of Microbiology, Rajah Muthiah Medical College, Annamalai University, Annamalai Nagar, Chidambaram-608 032, India

**Keywords:** *Asparagus racemosus*, Anticandidal activity, *Candida albicans*

## Abstract

The in vitro anticandidal activity of *Asparagus racemosus* roots and tubers extract was investigated against *Candida albicans, Candida tropicalis, Candida krusei, Candida guillermondii, Candida parapsilosis* and *Candida stellatoida,* which are isolated from vaginal thrush patients. The extract of *Asparagus racemosus* showed high degree of activity against all the *Candida* strains. The inhibitory effect of the extract against all the Candida tested was found comparable with that of standard antibiotics used.

*Asparagus racemosus*. Willd (Liliaceae) which is also known a *Shatavari,* is a herb employed in traditional medicine in many parts of the world. In India, *Asparagus racemosus* was most commonly used in Indigenous medicine[1]. *Asparagus racemosus* is recommended in Ayurvedic texts for prevention and treatment of gastric ulcers, dyspepsia and as a galactotgogue[2]. It is also used successfully for nervous disorders, inflammation, liver diseases and certain infectious diseases. The juice of fresh root of *Asparagus racemosus* has curative effect in patients with duodenal ulcers. Oral administration of decoction of powered root enhances the immuo-modulatory effect[3]. The present study was undertaken to evaluate the antifungal activity of *Asparagus racemosus* extract against *Candida* species.

The roots and tubers of *Asparagus racemosus* was collected in and around Chidambaram, Tamilnadu, India during January, 2005 and authenticated at the Herbarium, Department of Botany, Annamalai University, Annamalai Nagar. The roots and tubers were shade dried and powered. 25 g of powered plant samples were loaded in a Soxhelt apparatus and extracted in 125 ml of methanol. The extract so obtained was evaporated to dryness at 40-50° under vacuum. *Candida* strains were isolated from vaginal thrush patients, attending Obstetrics and Gynecology Department, Rajah Muthiah Medical College and Hospital, Annamalai Nagar, Tamilnadu, India and the species were identified using conventional tests.

The disc diffusion method was followed for antifungal susceptibility tests[4]. The 6 mm discs were impregnated with 20 μl of the extracts dissolved in 5% dimethylsulphoxide (DMSO) at the concentration of 25 mg/ml and placed on to inoculated Sabouraud's dextrose agar (SDA). Fluconazole discs at a concentration of 10 μg/disc were used as a positive control and 5% DMSO impregnated disc were used as negative control.

The two-fold serial dilution technique[5] with yeast nitrogen base was followed (5 to 0.312 mg/ml) for the determination of the minimum inhibitory concentration (MIC). The minimum fungicidal concentration (MFC) of the extracts was determined by plating 100 μl samples from each of MIC assay tube with growth inhibition into freshly prepared SDA plate. The plates were then incubated at 28° for 24-48 h. The MFC was recorded as the lowest concentration that did not permit any visible fungal colony growth on the agar plate after the period of incubation.

Results of screening of antifungal activity of *Asparagus racemosus* extract are summersed in Table 1. It is evident from the results, that the methanol extracts shows high anticandidal activity against all the *Candida* tested. The zone of inhibition ranged from 13 to 16 mm. The MIC values were between 2.5 to 0.312 mg/ml, while MFC values were between 5 to 0.625 mg/ml. The detailed chemical nature of the active principle(s) responsible for the antifungal activity is not known however, the preliminary screening has shown the presence of glycosides, steroids, saponins and flavonoids.

**TABLE 1:** ANTICANDIDAL ACTIVITY OF *ASPARAGUS RAMOSUS* EXTRACT

Microorganism	Diameter of Zone of Inhibition (mm)		Methanol extract mg/ml^c^	
		
	Methanol Extract^a^	Fluconazole^b^	MIC	MFC
*Candida albicans*	16	17	0.312	0.625
*Candida tropicalis*	16	18	0.625	1.25
*Candida krusei*	16	18	0.625	1.25
*Candida guillermondii*	16	18	0.625	1.25
*Candida parapsilosis*	14	18	0.625	1.25
*Candida stellatoida*	13	18	0.625	1.25

a The Diameter of zone of inhibition for the extract at the concentration of 25,000 μl/ml and

b Fluconazole at 10 μg/disc concentration.

c The Minimum Inhibitory concentration (MIC) and Minimum Fungicidal Concentration (MFC) of the extract against *Candid Sp*.
